# Utilization of *Carica papaya* latex on coating of SPIONs for dye removal and drug delivery

**DOI:** 10.1038/s41598-021-03328-2

**Published:** 2021-12-31

**Authors:** Antony V. Samrot, S. Saigeetha, Chua Yeok Mun, S. Abirami, Kajal Purohit, P. J. Jane Cypriyana, T. Stalin Dhas, L. Inbathamizh, S. Suresh Kumar

**Affiliations:** 1 School of Bioscience, Faculty of Medicine, Bioscience and Nursing, MAHSA University, 42610 Jenjarom, Selangor Malaysia; 2 Centre for Materials Engineering and Regenerative Medicine, Bharath Institute of Higher Education and Research, Chennai, Tamil Nadu 600073 India; 3 Department of Biotechnology, School of Bio and Chemical Engineering, Sathyabama Institute of Science and Technology, Rajiv Gandhi Salai, chennai, Tamil Nadu 600119 India; 4 Department of Microbiology, Kamaraj college, Tiruchendur Road, Thoothukudi, 628003 India; 5 Centre for Ocean Research, Earth Science and Technology Cell (ESTC), Sathyabama Institute of Science and Technology, Chennai, Tamil Nadu 600119 India

**Keywords:** Biotechnology, Nanobiotechnology, Plant biotechnology

## Abstract

Latex, a milky substance found in a variety of plants which is a natural source of biologically active compounds. In this study, Latex was collected from raw *Carica papaya* and was characterized using UV–Vis, FTIR and GC–MS analyses. Super Paramagnetic Iron Oxide Nanoparticles (SPIONs) were synthesized, coated with *C. papaya* latex (PL-Sp) and characterized using UV–Vis, FT-IR, SEM–EDX, XRD, VSM and Zeta potential analyses. SPIONs and latex coated SPIONs (PL-Sp) were used in batch adsorption study for effective removal of Methylene blue (MB) dye, where (PL-Sp) removed MB dye effectively. Further the PL-Sp was used to produce a nanoconjugate loaded with curcumin and it was characterized using UV–Vis spectrophotometer, FT-IR, SEM–EDX, XRD, VSM and Zeta potential. It showed a sustained drug release pattern and also found to have good antibacterial and anticancer activity.

## Introduction

Latex is usually stored in laticifers and is a sticky sap, exudes when a cut or wound is created. Latex has a variety of functions, including defensive characteristics and the repair of mechanical qualities in wounded plants^1–4^. Latex varies not just in chemical characteristics like alkaloids, terpenoids, proteins, phenols, and other phytochemical substances but also in colour^3^. Latex of papaya plants have high proteolytic capacity and they are utilised in a variety of industries, including cheese-making, meat tenderization, baking, and brewing/wine-making. It has a wide range of bioactivities including antioxidant, antibacterial, antiviral etc. and can be used to treat a wide range of diseases^5–7^.

Dyes like crystal violet, methylene blue, congo red etc. are detrimental to living beings and constitute a substantial threat to civilization due to their complex structures and non-biodegradable nature. Acidic dyes are harmful to the eyes, respiratory system, and skin, as well as having the potential to induce cancer and mutation in humans^8^. Adsorbents/catalysts to remove these are highly important, which can be obtained from biological sources^9^. SPIONs are much utilized nanoparticles for effective removal of these dyes^10^, the efficiency could be increased while coating with a biopolymer, where Samrot et al.^11^ found chitosan coated SPIONs to remove chromium efficiently than the naked SPIONs.

Cell membrane permeability, poor solubility of the encapsulated drug, substrate and by-product build up in the body are the key issues confronted by scientists in traditional drug delivery systems^12^. Biopolymers are structural component of several cells and tissues and naturally occurring molecules that contain either lengthy chains of proteins, lipids, nucleic acids, polysaccharides or combination of these biopolymers^13^. Poly lactic acid, polyhydroxyalkanoate and chitosan-based nanoparticles are reported to be delivering drugs very well^14–18^.

Having this basic information, in this study, papaya latex was collected, characterized and utilized to coat SPIONs (PL-Sp) and further PL-Sp was characterized. SPIONs and latex coated SPIONs were used in batch adsorption study where the adsorption parameters were optimized and isotherm was calculated for effective removal of methylene blue dye. Further the PL-Sp was used to produce a nanoconjugate loaded with the drug curcumin and it was characterized using various analytical techniques and various bioactivity studies were performed.

## Results and discussion

### Characterization of *Carica papaya* latex

All methods comply with local and national regulations. The latex was collected from raw papaya from our garden (Supplementary Fig. 1a,b,c,d). The absorbance maxima of aqueous extract was found at 270–280 nm and further no absorbance was recorded (Supplementary Fig. 2), which might be because of alkyl group present in latex^19,20^, where Samrot et al.^21^ reported *Calotropis gigantea* to show absorbance maxima at 228 nm. The crude latex showed peak near 3378 cm^−1^ and 3314 cm^−1^ indicated the -CH, -OH and -NH band stretching. The peak near 2939 cm^−1^ indicates the presence of –CH_3_ group. The bands near 1237 cm^−1^ indicated the presence of OH group (Supplementary Fig. 3). The peak near 830 cm^−1^–860 cm^−1^ indicated the presence of C–H bending^22^.

GC-MS analysis of aqueous extract of papaya latex revealed the presence of various bioactive compounds like 2-Hydroxy-gamma-butyrolactone, 1,3-propanediamine, hexadecanoic acid, octadecanoic acid etc. (Supplementary Fig. 4). Hexadecenoic acid was reported in red lady variety *C. papaya*^23^. Rf value for the *C. papaya* latex was found to be 0.836 when ethanol: water (2:1) was used as solvent system (Supplementary Fig. 5). Several metabolites with different Rf value were reported in unripe fruit of *C. papaya* L^24^.

### TLC-Bioautography for antioxidant activity

Yellow spot was identified in TLC plate at the Rf value of 0.836 (Supplementary Fig. 6) after spraying with DPPH where it confirmed its antioxidant property^25^. Latex of *Carica papaya* has been reported to antioxidant property^6, 7^.

### Characterization of SPIONs and Latex coated SPIONs

**Figure 1:**
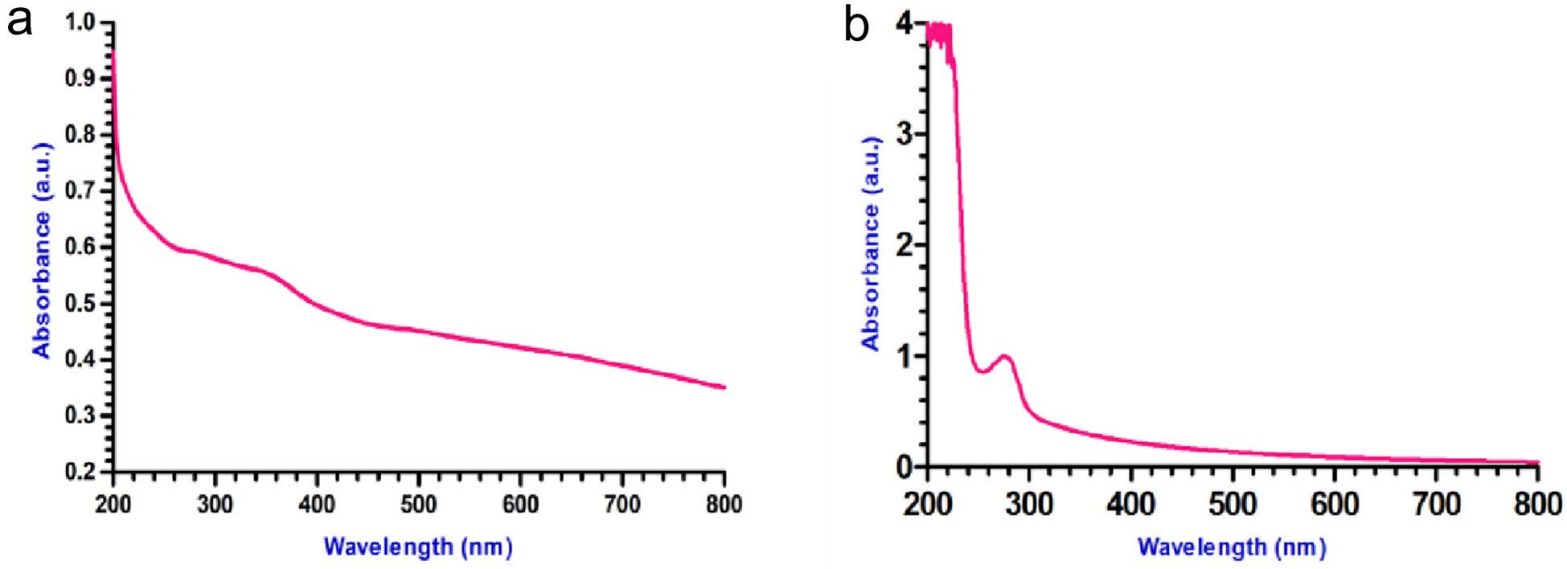
UV–Vis analysis of (**a**) SPIONs; (**b**) Latex coated SPIONs.

In UV–Vis Spectroscopy the adsorption maxima of the synthesized iron oxide nanoparticle were found to be at 260 to 340 nm (Fig. 1a) and for Latex coated SPIONs from 270 to 280 nm (Fig. 1b). This can be due to the presence of alkyl group present in the latex^20^. Samrot et al.^11^ synthesized SPIONs of which had absorption maxima around 260 nm.

**Figure 2:**
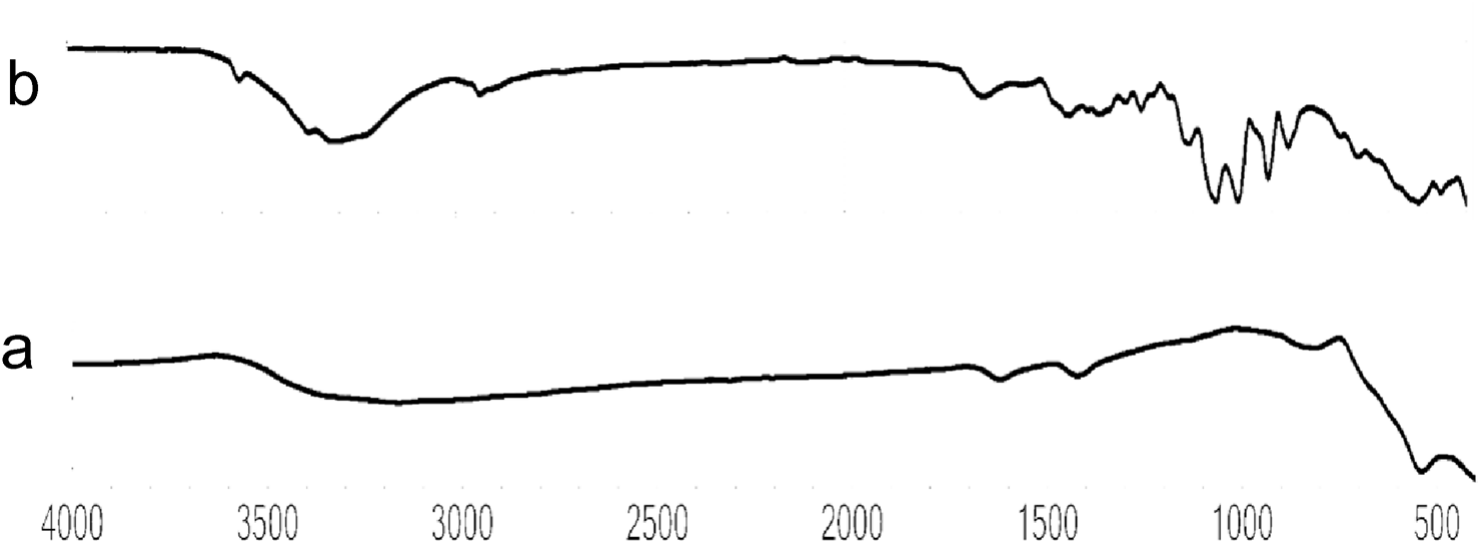
FTIR analysis of (**a**) SPIONs; (**b**) Latex coated SPIONs.

FT-IR spectra of SPIONs was found in the range of 569 cm^−1^ representing the stretching vibrations of Fe–O bond (Fig. 2a). Similar results were obtained by Samrot et al*.*^18^. The bands near 2921 cm^−1^, 1640 cm^−1^, 1458 cm^−1^ of papaya latex coated SPIONs indicated the presence of –CH, N– H, C– N– vibration of amino group. The band at 2925 cm^−1^ indicated the vibration of C–H stretching (Fig. 2b)^18,26^ where these functional groups were rendered by aqueous extract of papaya latex.

**Figure 3:**
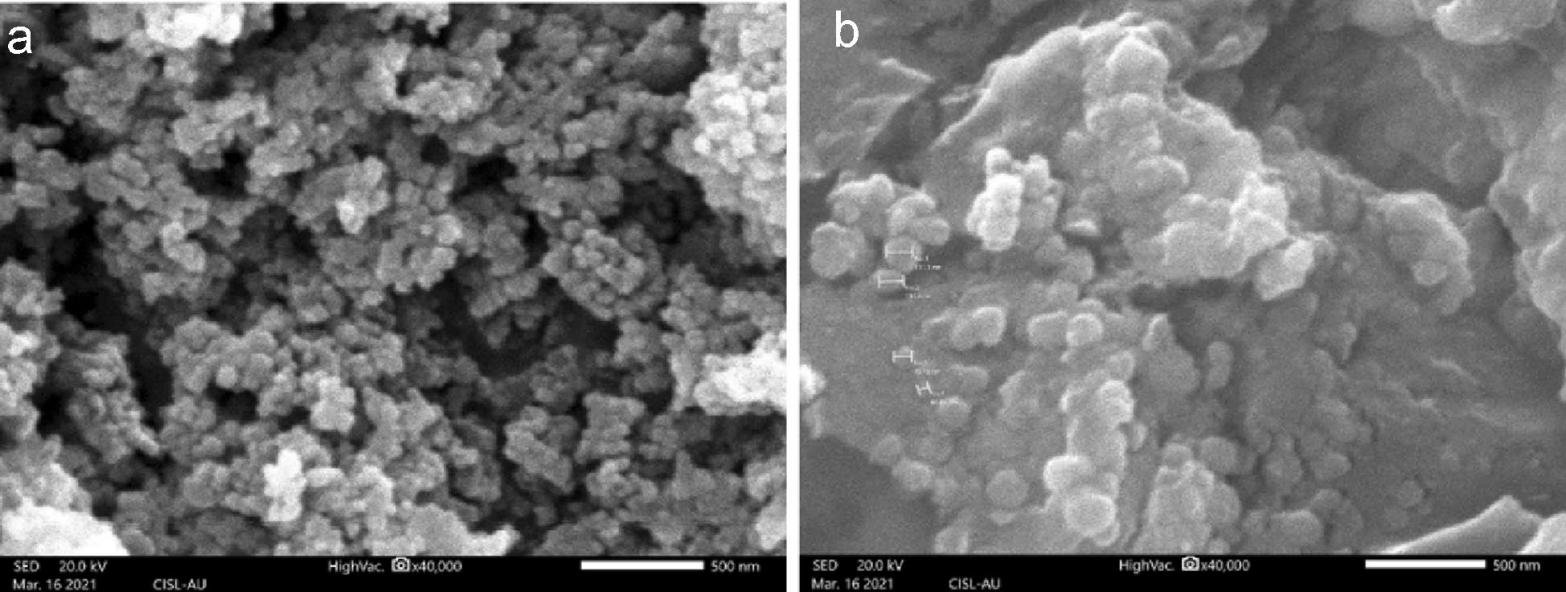
SEM analysis of (**a**) SPIONs; (**b**) Latex coated SPIONs.

SEM image showed that the synthesized SPIONs were in the size ranging between 30 and 60 nm (Fig. 3a). The SEM images showed that the papaya latex coated SPIONs were in the size ranging between 45 and 62 nm, the size increase might be due to coating with aqueous extract of *C. papaya* latex (Fig. 3b). Both the SPIONs and Latex coated SPIONs were spherical in shape. Samrot et al.^27^ produced SPIONs in the size of 25 nm whereas, Aghazadeh and Karimzadeh^28^, synthesized SPIONs in the size of 10 nm with no aggregation.

**Figure 4:**
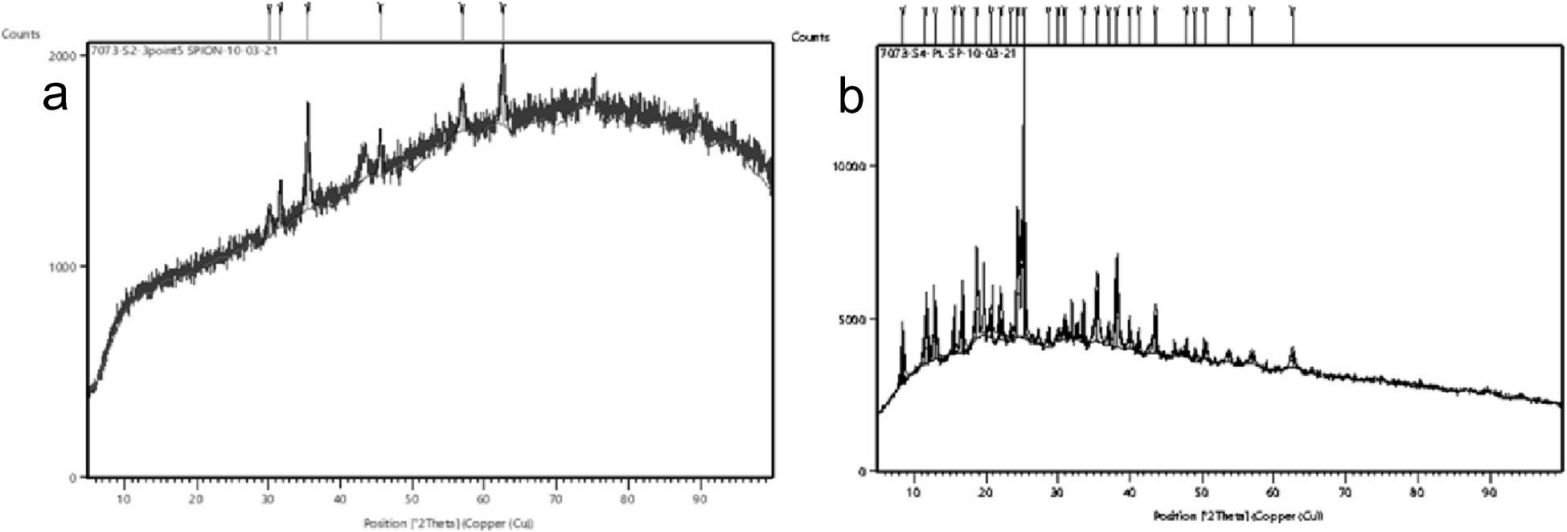
XRD analysis of (**a**) SPIONs; (**b**) Latex coated SPIONs.

The XRD patterns revealed that the synthesized SPIONs were crystalline in nature (Fig. 4a). The observed 2θ peaks at 30.1°, 31.6° 35.45°, 45.5°, 56.9°, 62.5° were in correspondence with the plane (220), (104), (311), (400), (511), (440) which referred to magnetite nanoparticle (JCPDS card no. 85–1436)^29^. The XRD patterns of latex coated SPIONs were too crystalline in structure and showed characteristic peaks of SPIONs at 2θ = 35.45°, 56.9°, 62.5°. Peaks at 12.9°, 18.7°, 20.6°, 23.4°, 28.6°, 35.4°, 41.1°, 49°, 56.9° were observed, which might be due to the presence of papain (Fig. 4b)^30,31^.

**Figure 5:**
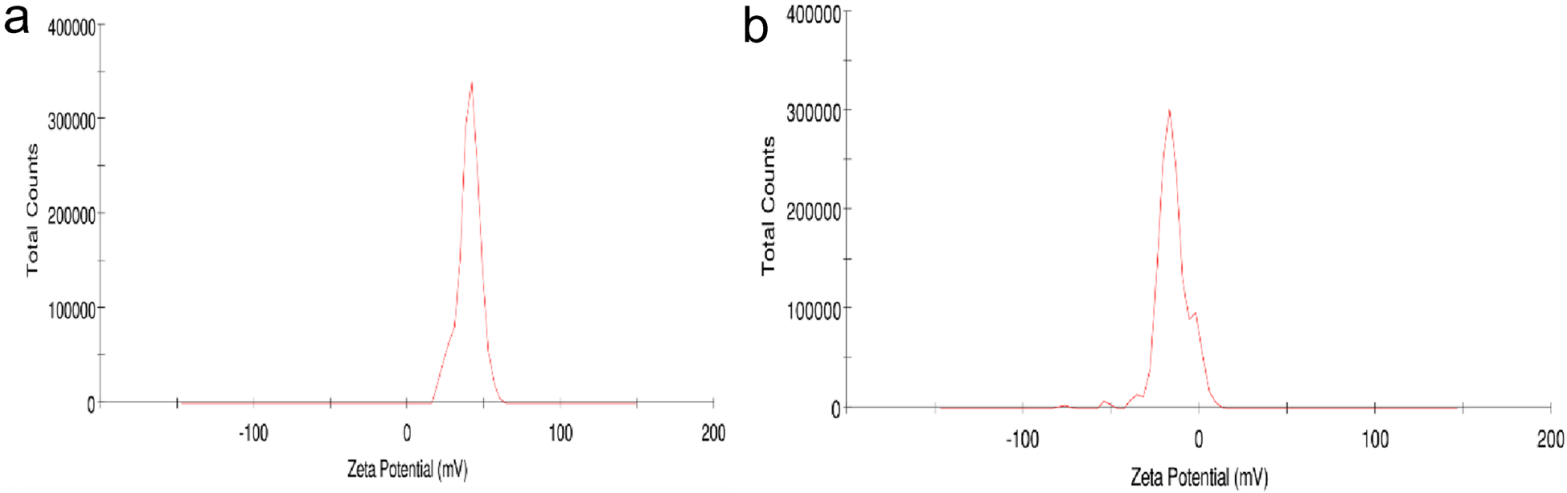
Zeta potential analysis of (**a**) SPIONs; (**b**) Latex coated SPIONs.

**Figure 6:**
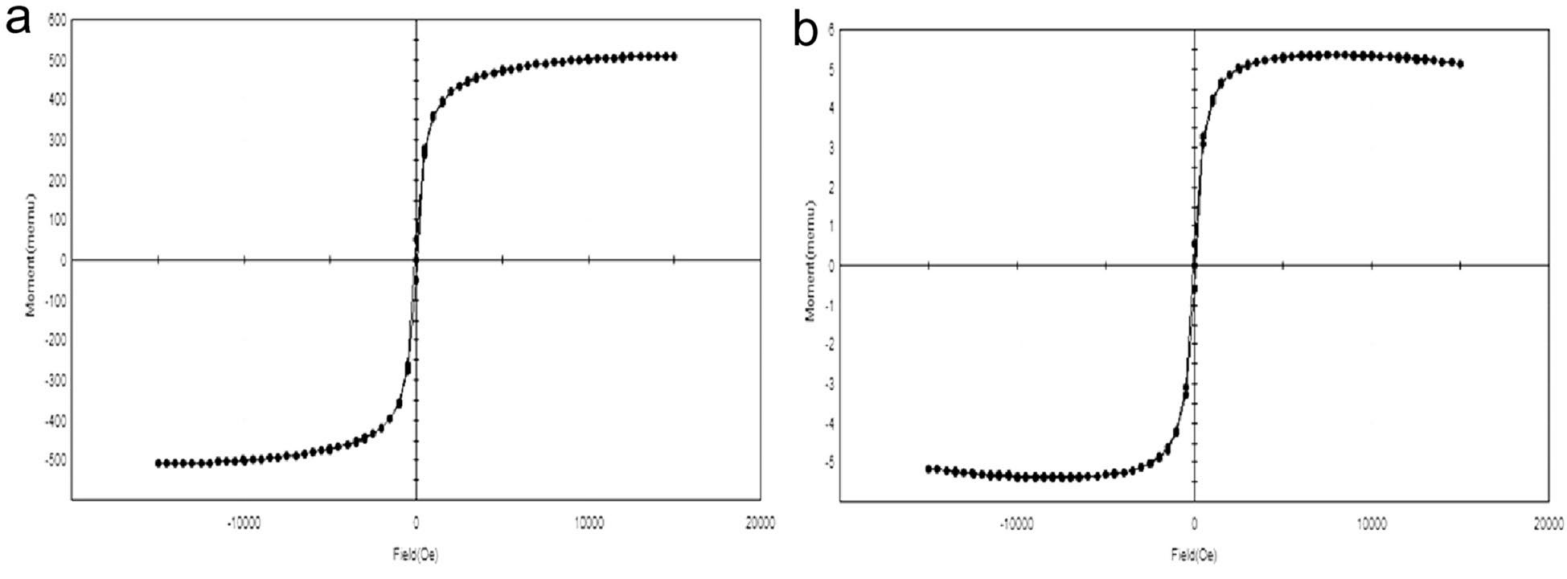
VSM of (**a**) SPIONs; (**b**) Latex coated SPIONs.

Zeta potential of the SPIONs and latex coated SPIONs was around 40.2 mV and − 15.2 mV respectively (Fig. 5a,b). Khatami et al.^32^ produced SPIONs which had zeta potential of 40.1 mV. The shift in the zetapotential was due to the coating with the aqueous extract of *C. papaya* and the coating made the SPIONs more stable. VSM of the synthesized SPIONs and Latex coated SPIONs were found to be superparamagnetic in nature as the magnetization increased around origin and no hysteresis loop was recorded (Fig. 6a,b). Mahmoudi et al.^33^ produced SPIONs with negligible remanence, coercivity in the hysteresis loop.

### Adsorption studies in batch systems

#### Optimization of adsorbate concentration

Here, fixed concentration of both the adsorbents SPIONs and Latex coated SPIONs was taken as 0.01 g and the concentration of methylene blue dye was varied from 1 to 10 ppm. The adsorbent and the adsorbate were allowed to interact for 1 h. 0.01 g of latex coated SPIONs was able to remove 10 ppm of MB dye with maximum removal efficiency of 80.01% (Supplementary Fig. 7). 40 mg of Magnetic nanoparticles (Fe_3_O_4_) were able to remove methylene blue dye of 70 ppm with removal efficiency of 89%^34^. SPIONs showed less removal efficiency compared with latex coated SPIONs (results not shown). Thus, latex coated SPIONs were subjected for further studies.

#### Optimization of adsorbent concentration

Here, the concentration of adsorbent which showed maximum removal from the aforementioned studies was varied from 0.01 to 0.1 g and it was allowed to interact for 1 h. In the above studies latex coated SPIONs showed maximum removal at 10 ppm hence, 10 ppm of dye was kept as constant and latex coated SPIONs was varied from 0.01 to 0.1 g. Here, 0.08 g of latex coated SPIONs was able to remove 10 ppm of dye with maximum removal efficiency of 98.75% (Supplementary Fig. 8). 8 g/l of SPIONs has been reported to remove 10 ppm of basic crystal violet dye with maximum removal of 94.7%^10^.

#### Optimization of pH

After optimizing adsorbate and adsorbent concentration, pH was optimized. pH was varied from 5 to 9 and maximum removal after interaction of 1 h was found to be 98.87% at pH 9 (Supplementary Fig. 9). pH is one of the most important factors to be optimized as ionic charge of adsorbent mostly depend on pH of the adsorbate solution. The functional groups present in the latex coated SPIONs are responsible for the binding of positively charged methylene blue dye at alkaline pH. Alizadeh et al.^35^ also reported the effective removal of crystal violet dye by fig and azola leaves coated with SPIONs.

#### Optimization of contact time

After the adsorbent-adsorbate and pH was optimized, contact time was varied in order to check how much interaction time was required to achieve maximum removal efficiency. 98.92% was removed at 3 h of interaction between the adsorbent and adsorbate at alkaline pH (Supplementary Fig. 10). Due to the increased surface area of the nanoparticle, it provided an active site for binding of MB dye till 3 h and further the removal percentage was found to decrease. Singh et al.^36^ used SPIONs-chitosan nanocomposite to remove oil from water and reported that the maximum removal efficiency was found to be till 3 h later the removal was found to be decreasing.

### Adsorption isotherm

**Figure 7:**
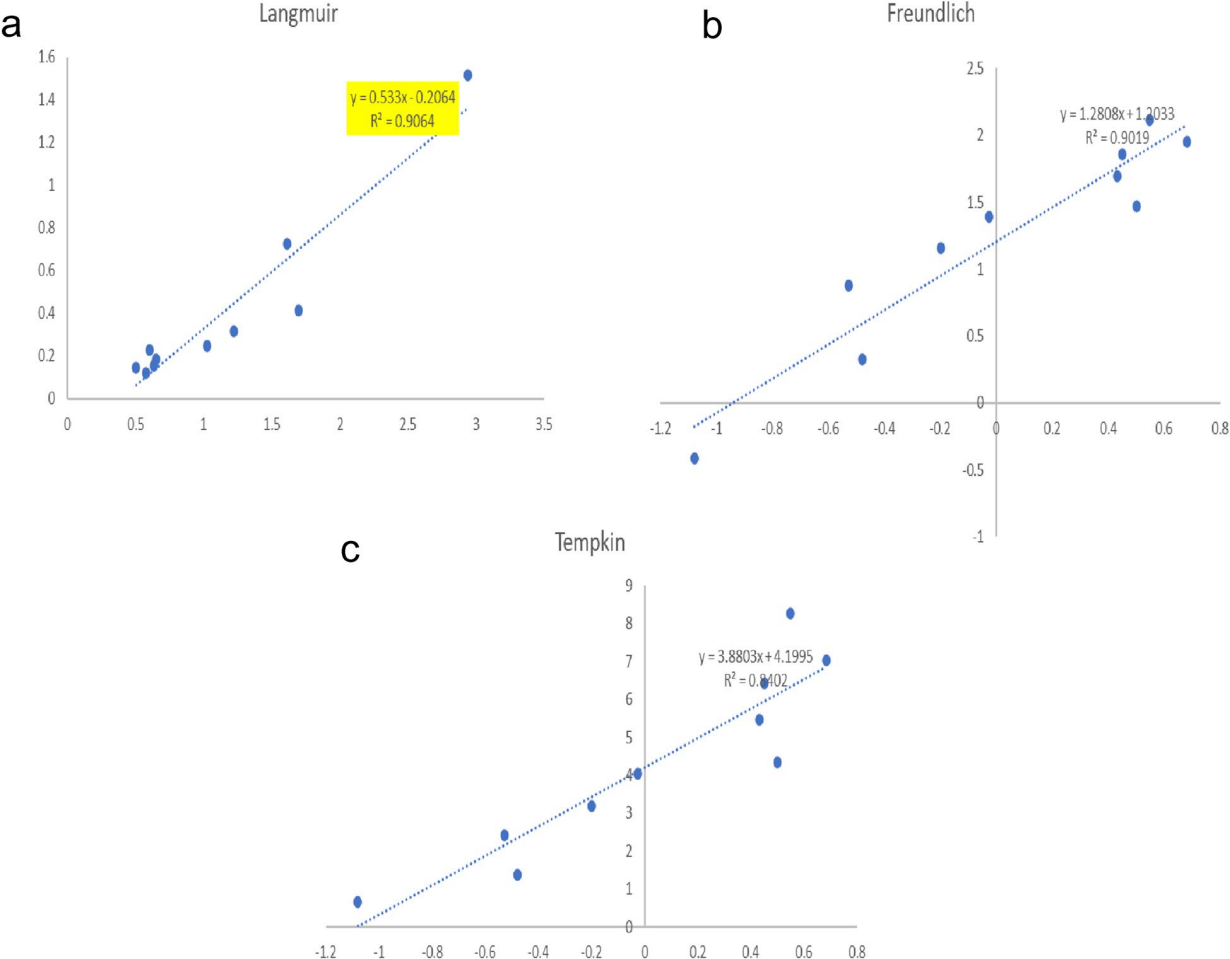
Adsorption Isotherm for methylene blue removal by latex coated SPIONs (**a**) Langmuir isotherm, (**b**) freundlich isotherm, (**c**) Tempkin isotherm.

R^2^ value is the correlation factor which helps us to determine the best fit isotherm model for the particular adsorption reaction. Here, adsorption isotherm was calculated by keeping the adsorbent (0.01 g SPIONs) as constant and adsorbate concentration (1–10 ppm) was varied. In this study the R^2^ value was 0.906, 0.901 and 0.84 for Langmuir, Freundlich and Tempkin isotherms respectively. Hence, the removal of MB dye by latex coated SPIONs followed Langmuir isotherm as the R^2^ value of the isotherm model was high compared to other isotherms (Fig. 7). Hosseinzadeh and Mohammadi^37^, also reported that anionic dyes were removed efficiently by magnetic iron oxide nanoparticles and followed Langmuir isotherm.

### Characterization of drug loaded nanoconjugate

UV–Vis of the synthesized drug loaded nanoconjugate was found to be around 260 nm and 290 nm, characteristics of SPIONs, curcumin and latex and further the absorbance found to be decreasing (Supplementary Fig. 11). Samrot et al.^38^ produced a drug loaded nanocarrier using *A. heterophylla* which showed maximum absorbance of 210 nm which indicated the presence of xylose content in polysaccharide.

FTIR was performed to identify the functional group present in the synthesized drug loaded nanoconjugate. The band near 1300 cm^−1^–1450 cm^−1^ indicated the presence of CH_3_ and CH_2_ bending^39^. The band near 1645 cm^−1^ indicated the presence of enolic CO group of the drug curcumin^40^. The band at 3450 cm^1^ confirmed the presence of hydroxyl group of curcumin in drug loaded nanocarrier (Supplementary Fig. 12)^41^.

**Figure 8:**
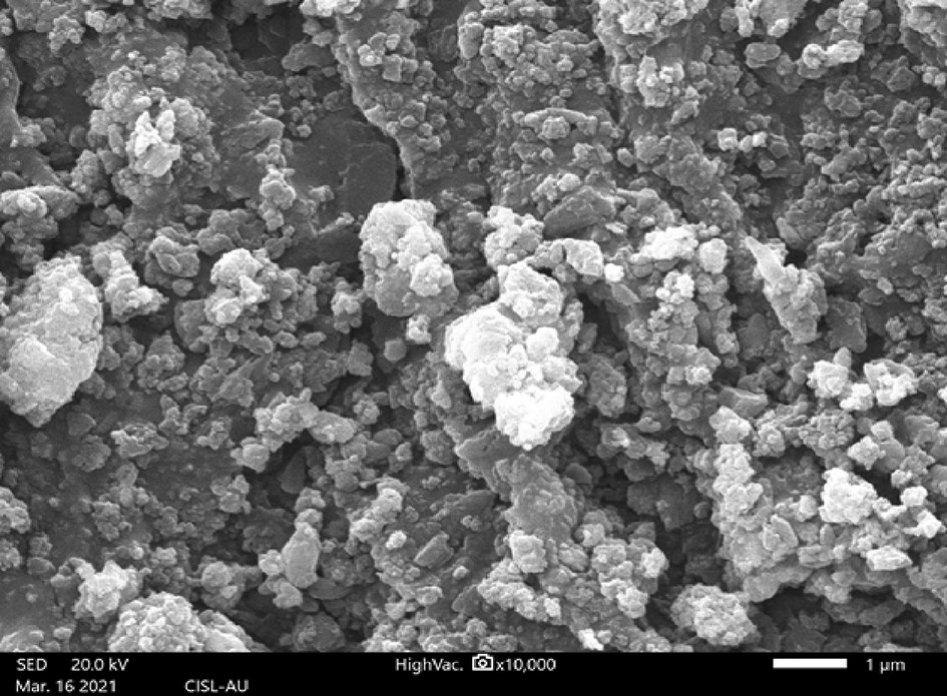
SEM of drug loaded nanoconjugate.

SEM analysis revealed that the surface of the drug loaded nanoconjugate was rough and was aggregated. The synthesized nanoconjugate was size around 111 nm in size (Fig. 8). The increase in size was due to the addition of drug curcumin. When SLS, CTAB, and SPAN 20 were used as surfactants, Pradeepkumar et al.^42^ was able to synthesize doxorubicin-loaded *C. gigantea* nanocarriers with a size around 100 nm.

The XRD patterns showed that the produced curcumin drug loaded nanoconjugates were slightly amorphous in nature. 2θ peaks at 56.9°, 62.5° were in correspondence with the plane (511), (440) which referred to magnetite nanoparticle^43^. Sharp intense peaks observed at 2θ = 29.7°, 35.3°, 53.3° where these shifts indicated the drug loading onto the nanoconjugate (Supplementary Fig. 13). Justin et al.^44^ was able to maintain the crystalline structure of the curcumin loaded SPIONs nanocarrier even after functionalization of SPIONs and final coating with chitosan.

Zeta potential analysis was performed and the charge of the drug loaded nanoconjugate was found to be − 23.6 mV (Supplementary Fig. 14). The values obtained by Samrot et al.^39^ for the drug loaded nanocarrier synthesized using carboxymethylated polysaccharides of *Terminalia catappa* and chelated using tri sodium tri meta phosphate was found to be − 38.06 mV. Vibrating sample magnetometer revealed that synthesized drug loaded nanoconjugate was superparamagnetic in nature. The magnetization power was retained around origin (Supplementary Fig. 15), thus the drug loading onto the nanoconjugate did not alter the magnetic property of SPIONs.

### Drug encapsulation efficiency

**Figure 9:**
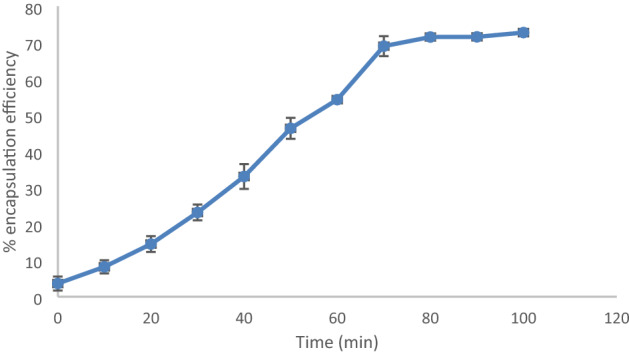
Percentage drug encapsulation efficiency of nanoconjugate.

The drug loaded nanoconjugate was analysed for its drug encapsulation efficiency and it was observed that the maximum encapsulation of the hydrophobic drug curcumin into the nanoconjugate was around 72.75% (Fig. 9). The percentage of encapsulation was found to be increasing as the time increased. At first, the curcumin got entrapped with the readily available sites in the latex coated SPIONs but as the time increased the curcumin entrapped in all the free spaces available. This might be the reason for increase in the encapsulation efficiency as the time increases. Shobana et al. (2019) reported a better encapsulation efficiency of 90% when curcumin was loaded into the STMP chelated chitosan nanoparticles.

### Drug release efficiency

**Figure 10:**
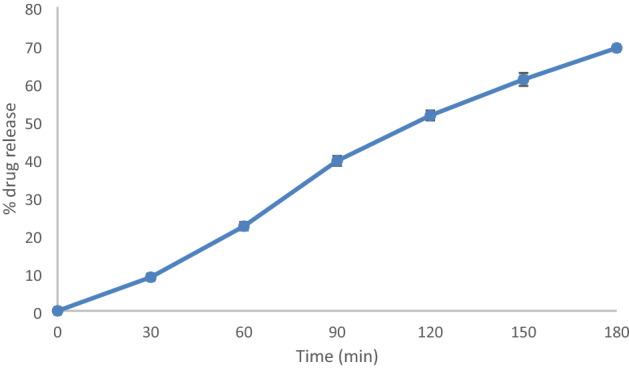
Percentage drug release efficiency of nanoconjugate.

Percentage drug release was calculated at every 30 min till 180th minute and the maximum drug release of 69.19% of the total encapsulated drug was observed at 180th minute. (Fig. 10). As the nanoconjugate was coated with aqueous extract of latex, which was getting dissolved when the solvent system PBS buffer was used, and the encapsulated drug was released. Curcumin release was reported to be till 240th minute by *Azadirachta indica* gum based nanocarrier^45^.

### Bioactivity studies

#### Antibacterial activity

**Figure 11:**
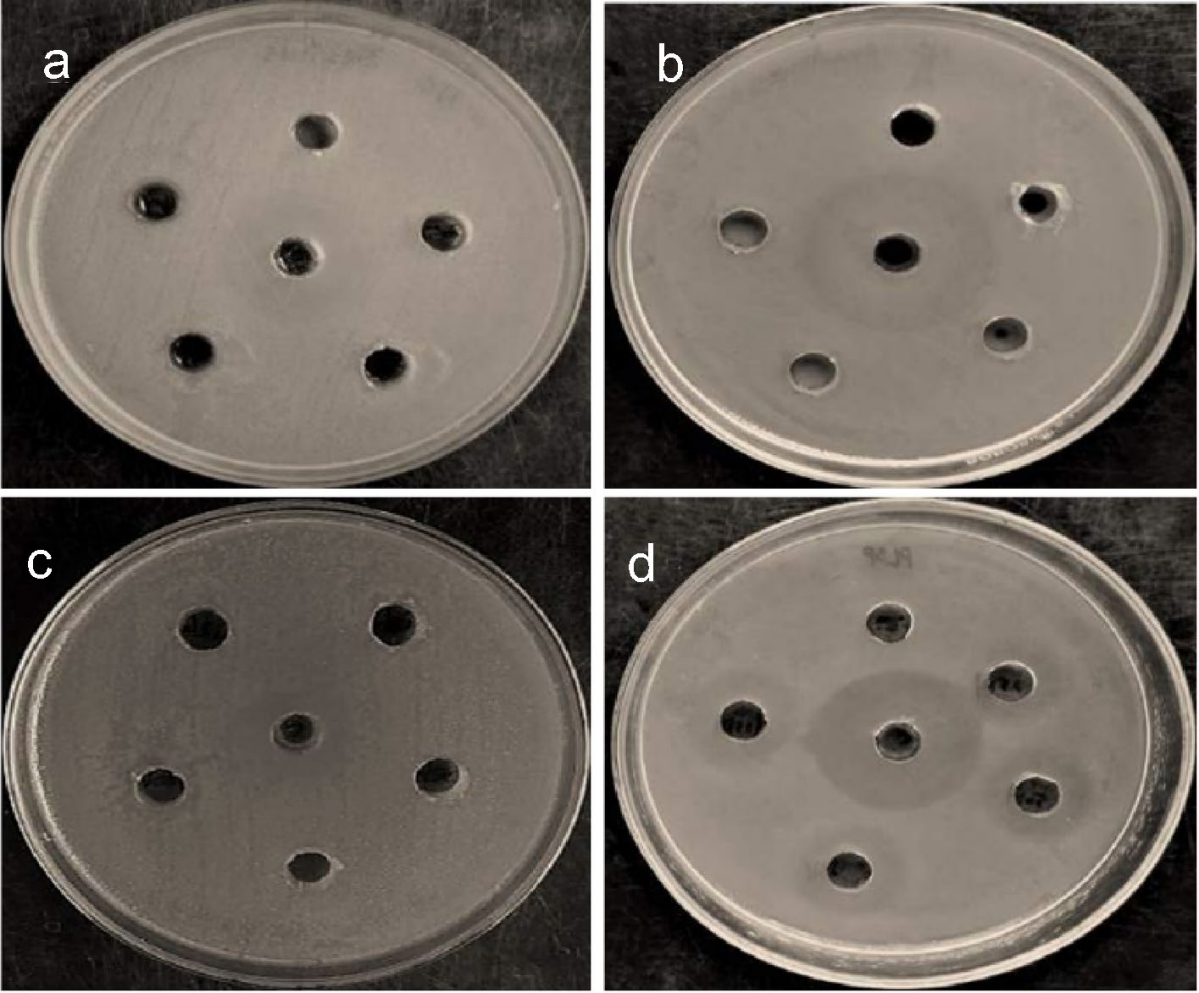
Antibacterial activity of (**a**) Latex coated SPIONs against *Bacillus subtilis,* (**b**) Latex coated SPIONs against *Pseudomonas aeruginosa,* (**c**) drug loaded nanoconjugate against *Bacillus subtilis,* (**d**) *Pseudomonas aeruginosa.*

SPIONs, *C*. *papaya* latex coated SPIONs and drug loaded nanoconjugate were tested for antibacterial activity against a Gram-positive bacterium (*Bacillus subtilis*) and a Gram-negative bacterium (*Pseudomonas aeruginosa*). SPIONs did not show any antibacterial activity (results not shown). Only curcumin loaded nanoconjugate showed inhibition against the Gram-negative bacterium at all the concentrations used (Fig. 11, Table. 1). This could be due to the release of curcumin. The growth of *P. gingivalis*, *Fusobacterium nucleatum*, *Prevotella intermedia*, and *Treponema denticola* was inhibited by curcumin in a dose-dependent manner^46^.

**Table 1:** Antibacterial activity of Latex coated SPIONs against *Bacillus subtilis* and *Pseudomonas aeruginosa.*

Name of the organism	Zone of Inhibition(mm)								ZOI (mm) control
Concentration	25 µg		50 µg		75 µg		100 µg		30 µg/well
	LSP	DSP	LSP	DSP	LSP	DSP	LSP	DSP	
*Bacillus subtilis*	–	–	–	–	–	–	–	2	21
*Pseudomonas aeruginosa*	–	4	–	5	–	6	–	10	28

*LSP* latex coated SPIONs, *DSP* drug loaded nanoconjugate.

#### Anticancer activity

##### MTT assay

**Figure 12:**
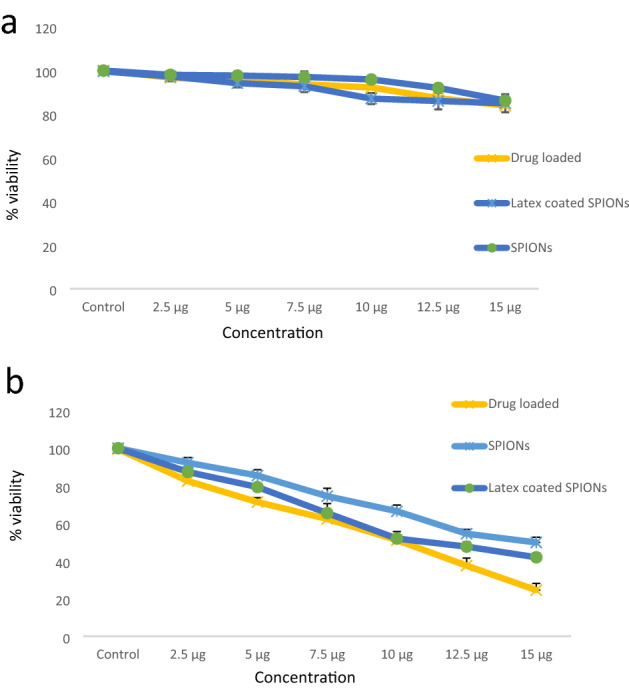
MTT assay against (**a**) normal fibroblast L929 cells (**b**) breast cancer MDA-MB-231 cell lines.

The SPIONs, latex coated SPIONs and drug loaded nanoconjugate did not show much activity on normal fibroblast L929 cell lines (Fig. 12a) where SPIONs, latex coated SPIONs and the curcumin loaded nanoconjugate had showed activity against the breast cancer cell lines where drug loaded nanoconjugate showed IC_50_ (inhibition concentration 50) at least concentration around 12 µg concentration (Fig. 12b). The enhanced activity was due to the release of curcumin by the nanoconjugate. Interestingly, cytotoxicity of drug loaded nanoconjugate was found against cancer cell line alone and did not show much inhibition on normal cells, thus, so it can be used in cancer therapy. Curcumin loaded nanoparticles have been reported be active against various cancer cell lines including PC3, MCF-7 etc^47^.

##### AO/EB staining

**Figure 13:**
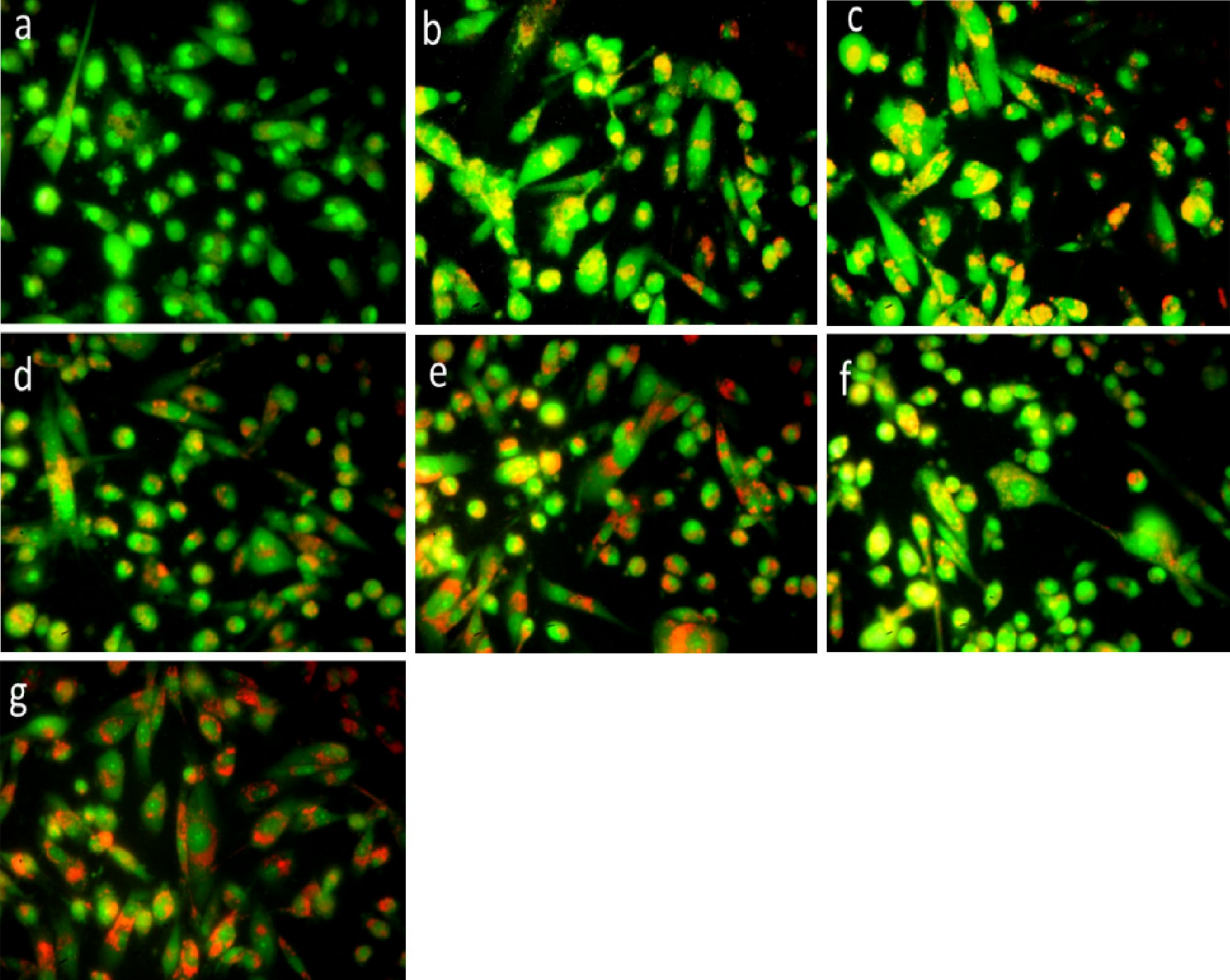
AO/ EB staining (**a**) Control; (**b**) 12.5 μg/ml SPIONs; (**c**) 15 μg/ml SPIONs; (**d**) 12.5 μg/ml papaya latex coated SPIONs; (**e**) 15 μg/ml papaya latex coated SPIONs; (**f**) 10 μg/ml drug loaded nanoconjugate; (**g**) 12.5 μg/ml drug loaded nanoconjugate.

Anticancer activity of SPIONs, latex coated SPIONs and drug loaded nanoconjugates were further confirmed by AO/EB staining. The apoptotic cells were found more in treated groups by observing the orange spot in the cells which was distinguished from the green stained live cells. Breast cancer MDA-MB-231 cells treated with sample SPIONs (12.5 µg/ml & 15 µg/ml), Latex coated SPIONs (12.5 & 15 µg/ml) and drug loaded nanoconjugate (10 & 12.5 µg/ml). Maximum apoptotic cells were found when drug loaded nanoconjugate (15 µg/ml) was used against MDA-MB-231 cell lines which indicated its high anticancer nature (Fig. 13a–g), this also due to the action of curcumin release by the nanoconjugate. Apoptotic and necrotic breast cancer cells were observed as red fluorescence due to their loss of membrane integrity when tamoxifen was used as drug in chitosan nanoparticles^48^.

## Materials and methods

### Materials

*Carica papaya* latex was collected from unripe papaya, Ferrous sulphate (SRL, India), Ferric chloride (SRL), Sodium hydroxide pellets (SRL, India), Ammonia solution (SRL, India), Hydrochloric acid (RANKEM, India), Methanol (RANKEM, India), Curcumin (SRL, India), Dialysis Bag (HIMEDIA, India), Methylene Blue (SRL, India) were used in this study. All the solvents and reagents used in this study were analytical grade. Nitrogen purged Milli Q water was used throughout the study.

### Collection of papaya latex

Latex of *Carica papaya* was collected from unripe raw papaya (Supplementary Fig. 1a,b,c). Latex was collected in a sterilized glass container (Supplementary Fig. 1d,e) and stored in refrigerator.

### Characterization of Carica papaya latex

Latex was dried and 10 g dissolved in 100 mL distilled water and was centrifuged at 5000 rpm, supernatant collected was subjected for characterization. UV–Vis spectrum range between 200 and 800 nm was recorded using UV–Visible spectroscopy (Shimadzu UV-1800, Japan). FTIR analysis for was performed (Shimadzu, Japan) to identify the functional group present in the latex. GC-MS (Shimadzu, QP2010 Plus) analysis was performed to identify the bioactive compounds present in the latex. Thin layer Chromatography was performed for the latex having ethanol: water (2:1) as mobile phase. Rf value was determined.

### TLC-bioautography for antioxidant activity

Thin layer Chromatography was performed for the latex as above mentioned. DPPH solution (0.004% (w/v) in 90% of methanol) was prepared and sprayed on TLC plates which was run with latex sample with aforementioned solvent system and checked for presence of yellow spot^25^.

### Synthesis of SPIONs

Super Paramagnetic Iron Oxide Nanoparticles were synthesized according to Samrot et al.^49^ with slight modification. Precursor salts 1 M Ferrous Chloride (FeCl_3_·6H_2_O) and 1 M Ferrous Sulphate (FeSO_4_·7H_2_O) was taken in 100 ml of nitrogen purged Milli Q water and was made as a homogenous solution by constant stirring. 150 ml of 1 M Sodium Hydroxide (NaOH) was prepared and it was added drop by drop to the precursor solution under constant stirring at 60 °C. 50 ml of ammonia solution was added drop by drop simultaneously to the solution until it turns black. The nanoparticles formed were collected by applying external magnetic field, washed several times with nitrogen purged Milli Q water. The pH was neutralized by washing with Milli Q water and it was freeze dried.

### Synthesis of latex coated SPIONs

Latex coated SPIONs was prepared following the modified method of Zhang et al.^50^. 10 g of latex was dissolved in 100 ml of nitrogen purged Milli Q water (10%) and centrifuged for 15 min at 3000 rpm and the supernatant collected was used for further coating. 100 mg of SPIONs was added into the extracted latex solution and was sonicated for 30 min. They were separated by applying external magnetic field. The obtained coated SPIONS were washed thrice with nitrogen purged Milli Q water and lyophilized.

### Characterization of SPIONs and latex coated SPIONs

Both the SPIONs and Latex coated SPIONs were characterized using UV-Vis Spectroscopy (Shimadzu, UV 3600 Plus), Fourier Transform Infrared Spectroscopy (Shimadzu, IRTRACER 100, Japan), X-Ray Diffraction Spectroscopy (PANalytical, Netherlands), Scanning Electron Microscopy (Carl Zeiss Ultra plus, Germany), Zeta Potential (ZETASIZER Nano Series ZSP) and Vibrating Sample Magnetometer (Lake Shore).

### Adsorption studies in batch experimental systems

#### Preparation of methylene blue (MB) dye solution

For this study, methylene blue (MB) was prepared at the concentration of 10 ppm. Different ppm of the dye solution was obtained by making further dilutions from the prepared 10 ppm of stock solution. The dye solution was taken in an aliquot and measured spectroscopically at 663 nm (Results not shown). A graph was plotted with concentration in X-axis and optical density (OD) in Y-axis.

#### Optimization of adsorbate concentration-best adsorbent identification

0.01 g of SPIONs and Latex coated SPIONs was taken as adsorbent materials and was kept as constant and the ppm concentration of MB was varied from 1 to 10 ppm. Adsorbate and the adsorbent were allowed to interact for 1 hour. Later the mixture was centrifuged at 5000 rpm for 15 min and the supernatant was collected and it was measured spectroscopically at 663 nm. Removal percentage was calculated from the standard curve. The % removal was calculated from the following formula

$$\% Removal = \frac{{\left( {initial\; concentration - final\; concentration} \right)}}{initial\; concentration} \times 100$$

A graph was plotted by having concentration in terms of ppm in X-axis and % removal in Y-axis. The adsorbent material which showed highest percentage of removal was taken for the further optimization studies.

#### Optimization of adsorbent concentration

Best adsorbent was identified from the previous study and now keeping the adsorbate concentration in which, it showed maximal removal as constant and the concentration of the best adsorbent was varied from 0.01 to 0.1 g and it was allowed to interact with the adsorbate for 1 h. Later, the mixture was centrifuged at 5000 rpm for 15 min and the supernatant was collected and it was measured spectroscopically at 663 nm. Removal was calculated from the standard curve. A graph was plotted by having adsorbent concentration in X-axis and % removal in Y-axis.

#### Optimization of pH

After the optimization of adsorbent and adsorbate concentration, pH was optimized in order to enhance the removal efficiency. pH was varied from 5 to 9 and it was altered using 0.1 N NaOH and 1 N HCl. It was allowed to interact and centrifuged at 5000 rpm for 15 min. Supernatant was collected and measured spectroscopically at 663 nm. Removal percentage was calculated from the standard curve. A graph was plotted by having pH in X-axis and % removal in Y-axis.

#### Optimization of contact time

Contact time was further optimized after optimizing adsorbent-adsorbate concentration and pH. Adsorbent and adsorbate (optimized) were allowed to interact in an optimized pH in varied contact hours (1–5 h). After the end of each hour the solution was centrifuged at 5000 rpm for 15 min. Supernatant was collected and measured spectroscopically at 663 nm. Removal percentage was calculated from the standard curve. A graph was plotted by having Contact time in terms of hours in X-axis and % removal in Y-axis.

### Adsorption isotherms

The mechanism taking place between adsorbent and the adsorbate was studied using adsorption isotherm. Here, adsorption isotherm was calculated by keeping the adsorbent as constant and adsorbate concentration was varied. ﻿Adsorption capacity at equilibrium qe= (Ci-Ce)V/m,  Ci and Ce are the initial and final concentration; V and mM are the volume of the adsorbate and mass of the adsorbent respectively. ﻿Langmuir (1/qe vs. 1/Ce); Freundlich (ln qe vs. ln Ce) and Tempkin (qe vs. ln Ce) isotherm models were calculated.

### Production of drug loaded nanoconjugate using latex coated SPIONs

Nanoconjugate was produced by slight modification of Akbarian et al.^51^. 0.2 g of latex coated SPIONs was taken and was added to 100 ml of nitrogen purged Milli Q water and sonicated for 10 min. 0.01 g of drug (curcumin) was added to 10 ml of non-polar solvent methanol and mixed. Methanol containing curcumin was added to the latex coated SPIONs solution and was sonicated for 30 min. Later, the solution was centrifuged at 5000 rpm for 20 min and the pellet was collected. The mixture was washed thrice and lyophilized.

### Characterization of drug loaded nanoconjugate

Drug loaded nanoconjugate was characterized using UV–Vis Spectroscopy (Shimadzu, UV 3600 Plus), Fourier Transform Infrared Spectroscopy (Shimadzu, IRTRACER 100, Japan), X-Ray Diffraction Spectroscopy (PANalytical, Netherlands), Scanning Electron Microscopy-(Carl Zeiss Ultra plus, Germany), Zeta Potential (ZETASIZER Nano Series ZSP) and Vibrating Sample Magnetometer (Lake Shore).

### Drug encapsulation efficiency

After loading the drug curcumin, the nanoconjugates were centrifuged at 7000 rpm for 15 min, 1 ml of supernatant was collected and subjected to UV–Vis spectroscopy at 427 nm. Drug encapsulation efficiency was performed as reported^39,52^. Graph was plotted by taking time in the X-axis and % drug encapsulation in Y axis.

### Drug release efficiency

10 mg of synthesized drug loaded nanoconjugate were dissolved in 1 mL of nitrogen purged Milli Q water. Phosphate buffer solution (PBS) of pH 7.4 (Sodium chloride—8 g, Potassium chloride—200 mg, Disodium hydrogen phosphate—1.44 g, Potassium dihydrogen phosphate—245 mg) was prepared. The nanoconjugates were put in dialysis bag and dialyzed against phosphate buffer with slight modifications in the composition of PBS and its pH^41^. Absorbance was measured by taking the OD values at 30 min interval. % Drug release was calculated at every 30th minute using the formula^53^

$$\% Drug\; release = (Drug\; release\; at\; time\;{\text{"t"}}/{\text{ Encapsulated}} \;drug) \times 100$$

Graph was plotted by taking time in the X-axis and % drug release in Y axis.

### Bioactivity studies

SPIONs, Latex coated SPIONs, Drug loaded nanoconjugate were checked for its antibacterial activity-agar well diffusion method^54^ and anticancer activity against normal fibroblast L929 and breast cancer MDA-MB-231 cell lines (procured from National Centre for Cell Sciences (NCCS), Pune, India) was performed using MTT assay^55^ and AO/ EB staining^56^.

### Statistical analysis

Triplicates were performed for all the experiments and all the value are given as mean ± standard deviation value here.

### Graphical abstract

The complete work is given in terms of graphical abstract (Supplementary Fig. 16).

## Conclusion

*C. papaya* L latex was collected and characterized. The aqueous extract was utilized to coat Super paramagnetic iron oxide nanoparticles (SPIONs), then characterized and utilized for various application including dye removal and drug delivery. In batch adsorption studies, latex coating onto SPIONs was enhancing the removal efficiency of methylene blue dye, 10 ppm of MB dye was removed by 0.08 g of latex coated SPIONs with maximum removal of 98.92% at alkaline pH (pH 9) after 3 h of interaction time and it obeyed Langmuir isotherm with maximum R^2^ value of 0.906. Curcumin loaded nanoconjugate was produced and it showed a sustain drug release pattern. The drug loaded nanoconjugate showed better antibacterial activity and it also had excellent anticancer activity against breast cancer cell line. From this study, it is clear that *C. papaya* L latex can be used for SPIONs coating can be used for dye removal and drug delivery.

## Supplementary Information

Supplementary Information.

## Data Availability

The datasets used and/or analysed during the present study are available from the corresponding author on reasonable request.

## References

[1] Hunter JR (1994). Reconsidering the functions of latex. Trees.

[2] Dussourd DE, Eisner T (1987). Vein-cutting behavior: Insect counterploy to the latex defense of plants. Science.

[3] Agrawal AA, Konno K (2009). Latex a model for understanding mechanisms, ecology, and evolution of plant defense against herbivory. Annu. Rev. Ecol. Evol. Syst..

[4] Bauer G, Speck T (2012). Restoration of tensile strength in bark samples of Ficus benjamina due to coagulation of latex during fast self-healing of fissures. Ann. Bot..

[5] Hafid K, John J, Sayah TM, Domínguez R, Becila S, Lamri M, Gagaoua M (2020). One-step recovery of latex papain from Carica papaya using three phase partitioning and its use as milk-clotting and meat-tenderizing agent. Int. J. Biol. Macromol..

[6] Bandasak C, Rawdkuen S, Pintathog P, Chaiwut P (2011). Bioactivities of Carica papaya latex. Thai J. Agric. Sci.

[7] Saeed F, Arshad MU, Pasha I, Naz R, Batool R, Khan AA, Nasir MA, Shafique B (2014). Nutritional and phyto-therapeutic potential of papaya (*Carica Papaya* Linn.): An overview. Int. J. Food Prop..

[8] Sabnis RW (2010). Handbook of Biological Dyes and Stains Synthesis and Industrial Applications.

[9] Khin MM, Nair AS, Babu VJ, Murugan R, Ramakrishna S (2012). A review on nanomaterials for environmental remediation. Energy Environ. Sci..

[10] Samrot AV, Ali HH, Selvarani J, Faradjeva E, Raji P, Prakash P (2021). Adsorption efficiency of chemically synthesized Superparamagnetic Iron Oxide Nanoparticles (SPIONs) on crystal violet dye. CRGSC.

[11] Samrot AV, Sahithya CS, Selvarani J, Pachiyappan S (2019). Surface-engineered super-paramagnetic iron oxide nanoparticles for chromium removal. Int. J. Nanomed..

[12] Gopi S, Amalraj A, Thomas S (2016). Effective drug delivery system of biopolymers based on nanomaterials and hydrogels-a review. Drug Des..

[13] Vandamme, E., De Baets, S. & Steinbuchel, A. in *Biopolymers*. (ed. Steinbuchel, A.), (series editor). (Wiley, 2004).

[14] Jayakumar R, Menon D, Manzoor K, Nair SV, Tamura H (2010). Biomedical applications of chitin and chitosan based nanomaterials—A short review. Carbo. Pol..

[15] Senthilkumar P, Dawn SS, Samanvitha KS, Kumar SS, Kumar GN, Samrot AV (2017). Optimization and characterization of poly[R]hydroxyalkanoate of Pseudomonas aeruginosa SU-1 to utilize in nanoparticle synthesis for curcumin delivery. BCAB.

[16] Senthilkumar P, Dawn SS, Samanvitha SK, Saipriya C, Samrot AV (2019). Surfactant Mediated Synthesis of Polyhydroxybutyrate (PHB) Nanoparticles for Sustained Drug Delivery.

[17] Shobana N, Kumar PS, Raji P, Samrot AV (2019). Utilization of crab shell-derived chitosan in nanoparticle synthesis for Curcumin delivery. IJMS.

[18] Samrot AV, Burman U, Philip SA, Shobana N, Chandrasekaran K (2018). Synthesis of curcumin loaded polymeric nanoparticles from crab shell derived chitosan for drug delivery. Inform. Med. Unlocked.

[19] Maisarah, A. M., Nurul Amira, B., Asmah, R. & Fauziah, O. Antioxidant analysis of different parts of Carica papaya. *Int. Food Res. J* ***20***(3), (2013)

[20] Pawar RP (2016). Separation and identification of active constituents of calotropis gigantea latex, by HPLC, FTIR, UV-visible and classical techniques. World J. Pharm. Life Sci..

[21] Samrot AV, Sahiti K, Bhavya KS, Suvedhaa B (2019). Synthesis of plant latex based hybrid nanocarriers using surfactants for curcumin delivery. J. Clust. Sci.

[22] Spanò D, Pintus F, Mascia C, Scorciapino MA, Casu M, Floris G, Medda R (2012). Extraction and characterization of a natural rubber from Euphorbia characias latex. Biopolymers.

[23] Gorane A, Naik A, Nikam T, Tripathi T, Ade A (2018). GCMS analysis of phytocomponents of C. papaya variety red lady. J. Pharmacogn. Phytochem.

[24] Varsha A, Husain AS, Javed NK, Poonam A (2013). Physico-chemical and phytochemical evaluation of Cpapaya Linn unripe fruits. Int. Res. J. Pharm..

[25] Samrot AV, Bennet Rohan D, Kumar D, Sahiti K, Raji P, Sree Samanvitha K (2016). Detection of antioxidant and antibacterial activity of Mangifera indica using TLC bio-autography. Int. J. Pharm. Sci. Res..

[26] Seoudi R, Fouda AA, Elmenshawy DA (2010). Synthesis, characterization and vibrational spectroscopic studies of different particle size of gold nanoparticle capped with polyvinylpyrrolidone. Physica B.

[27] Samrot, A.V., Sai Bhavya, K., Sruthi P. D. & Paulraj, P. Synthesis of SPIONs to deliver drug in-vitro and to use as contrasting agent. *(IJARET)* ***11***(2) (2020b).

[28] Aghazadeh M, Karimzadeh I (2018). One-pot electro-synthesis and characterization of chitosan capped superparamagnetic Iron oxide nanoparticles (SPIONs) from ethanol media. Curr. Nanosci..

[29] Sruthi PD, Sahithya CS, Justin C, SaiPriya C, Bhavya KS, Senthilkumar P, Samrot AV (2018). Utilization of chemically synthesized super paramagnetic iron oxide nanoparticles in drug delivery, imaging and heavy metal removal. J. Clust. Sci..

[30] Drenth J, Jansonius JN, Koekoek R, Wolthers BG (1971). The structure of papain. Adv. Protein Chem..

[31] Liu Z, Li D, Dai H, Huang H (2017). Preparation and characterization of papain embedded in magnetic cellulose hydrogels prepared from tea residue. J. Mol. Liq..

[32] Khatami M, Alijani HQ, Fakheri B, Mobasseri MM, Heydarpour M, Farahani ZK, Khan AU (2019). Super-paramagnetic iron oxide nanoparticles (SPIONs) Greener synthesis using Stevia plant and evaluation of its antioxidant properties. J. Clean. Prod.

[33] Mahmoudi M, Simchi A, Imani M, Shokrgozar MA, Milani AS, Häfeli UO, Stroeve P (2010). A new approach for the in vitro identification of the cytotoxicity of superparamagnetic iron oxide nanoparticles. Colloids Surf. B.

[34] Dagher S, Soliman A, Ziout A, Tit N, Hilal-Alnaqbi A, Khashan S, Qudeiri JA (2018). Photocatalytic removal of methylene blue using titania-and silica-coated magnetic nanoparticles. Mater. Res. Express.

[35] Alizadeh N, Shariati S, Besharati N (2017). Adsorption of crystal violet and methylene blue on azolla and fig leaves modified with magnetite iron oxide nanoparticles. Int. J. Environ. Res..

[36] Singh H, Jain A, Kaur J, Arya SK, Khatri M (2020). Adsorptive removal of oil from water using SPIONs–chitosan nanocomposite kinetics and process optimization. Appl. Nanosci..

[37] Hosseinzadeh H, Mohammadi S (2016). Biosorption of anionic dyes from aqueous solutions using a novel magnetic nanocomposite adsorbent based on rice husk ash. Sep. Sci. Technol..

[38] Samrot AV, Kudaiyappan T, Bisyarah U, Mirarmandi A, Faradjeva E, Abubakar A, Subbiah SK (2020). Extraction, purification, and characterization of polysaccharides of Araucaria heterophylla L and Prosopis chilensis L and utilization of polysaccharides in nanocarrier synthesis. Int. J. Nanomed..

[39] Samrot AV, Suvedhaa B, Sahithya CS, Madankumar A (2018). Purification and utilization of gum from *Terminalia catappa* L. for synthesis of curcumin loaded nanoparticle and its in vitro bioactivity studies. J. Clust. Sci..

[40] Athira GK, Jyothi AN (2014). Preparation and characterization of curcumin loaded cassava starch nanoparticles with improved cellular absorption. Int. J. Pharm. Pharm..

[41] Samrot AV, Jahnavi T, Padmanaban S, Philip SA, Burman U, Rabel AM (2016). Chelators influenced synthesis of chitosan–carboxymethyl cellulose microparticles for controlled drug delivery. Appl. Nanosci..

[42] Pradeepkumar P, GovindarajaJeyaraj M, Munusamy AM, Rajan M (2017). Supplementary material: Assembling of multifunctional latex-based hybrid nanocarriers from Calotropis gigantea for sustained (doxorubicin) DOX releases. Biomed. Pharmacother..

[43] Ghandoor HE, Zida HM, Khalil MHH, Ismail MIM (2012). Synthesis and some physical properties of magnetite (Fe3O4) nanoparticles. Int. J. Electrochem. Sci..

[44] Justin C, Samrot AV, Sahithya CS, Bhavya KS, Saipriya C (2018). Preparation, characterization and utilization of coreshell super paramagnetic iron oxide nanoparticles for curcumin delivery. PLoS ONE.

[45] Samrot AV, Angalene JLA, Roshini SM, Stefi SM, Preethi R, Raji P (2020). Purification, characterization and exploitation of Azadirachta indica gum for the production of drug loaded nanocarrier. Mater. Res. Express.

[46] Izui S, Sekine S, Maeda K, Kuboniwa M, Takada A, Amano A, Nagata H (2016). Antibacterial activity of curcumin against periodontopathic bacteria. J. Periodontol..

[47] Anitha A, Deepagan VG, Rani VD, Menon D, Nair SV, Jayakumar R (2011). Preparation, characterization, in vitro drug release and biological studies of curcumin loaded dextran sulphate–chitosan nanoparticles. Carbohyd. Polym..

[48] Vivek R, Babu VN, Thangam R, Subramanian KS, Kannan S (2013). pH-responsive drug delivery of chitosan nanoparticles as Tamoxifen carriers for effective anti-tumor activity in breast cancer cells. Colloids Surf. B.

[49] Samrot AV, SaiPriya C, Selvarani J, Lavanya Y, Soundarya P, Varghese RJ (2020). A study on influence of superparamagnetic iron oxide nanoparticles (SPIONs) on green gram (Vigna radiata L.) and earthworm (Eudrilus eugeniae L.). Mater. Res. Express.

[50] Zhang L, Yu F, Cole AJ, Chertok B, David AE, Wang J, Yang VC (2009). Gum arabic-coated magnetic nanoparticles for potential application in simultaneous magnetic targeting and tumor imaging. AAPS J..

[51] Akbarian M, Mahjoub S, Elahi SM, Zabihi E, Tashakkorian H (2020). Green synthesis, formulation and biological evaluation of a novel ZnO nanocarrier loaded with paclitaxel as drug delivery system on MCF-7 cell line. Colloids Surf. B Biointerfaces.

[52] Dodi G, Pala A, Barbu E, Peptanariu D, Hritcu D, Popa MI, Tamba BI (2016). Carboxymethyl guar gum nanoparticles for drug delivery applications: Preparation and preliminary in-vitro investigations. Mater. Sci. Eng., C.

[53] Purushothaman BK, Maheswari PU, Begum KMS (2019). Magnetic assisted curcumin drug delivery using folate receptor targeted hybrid casein-calcium ferrite nanocarrier. J. Drug Deliv. Sci. Technol..

[54] Samrot AV, Raji P, Selvarani AJ, Nishanthini P (2018). Antibacterial activity of some edible fruits and its green synthesized silver nanoparticles against uropathogen–Pseudomonas aeruginosa SU 18. Biocatal. Agric. Biotechnol..

[55] Mosmann T (1983). Rapid colorimetric assay for cellular growth and survival application to proliferation and cytotoxicity assays. J. Immunol. Methods.

[56] Baskić D, Popović S, Ristić P, Arsenijević NN (2006). Analysis of cycloheximide-induced apoptosis in human leukocytes Fluorescence microscopy using annexin V/propidium iodide versus acridin orange/ethidium bromide. Cell Biol. Int.

