# Supplementary material for: Utilization of Carica papaya latex on coating of SPIONs for dye removal and drug delivery
Source: Sci Rep. 2021 Dec 31;11:24511. doi: 10.1038/s41598-021-03328-2 (PMC8720089; doi:10.1038/s41598-021-03328-2)
Supplement: Supplementary file 1 — Supplementary Information. [file 41598_2021_3328_MOESM1_ESM.docx]

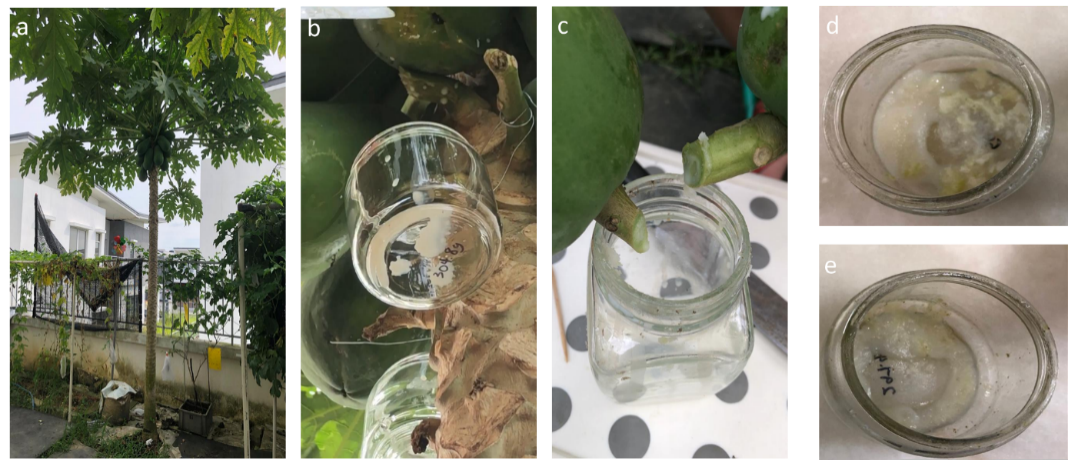


Supplementary fig. **1:** a) *Carica papaya* tree b) Collection of latex from unripe fruit skin, c) Collection of latex from unripe papaya, d) and e) Storage of latex in glass container

Supplementary fig. 2. UV- Vis of Latex


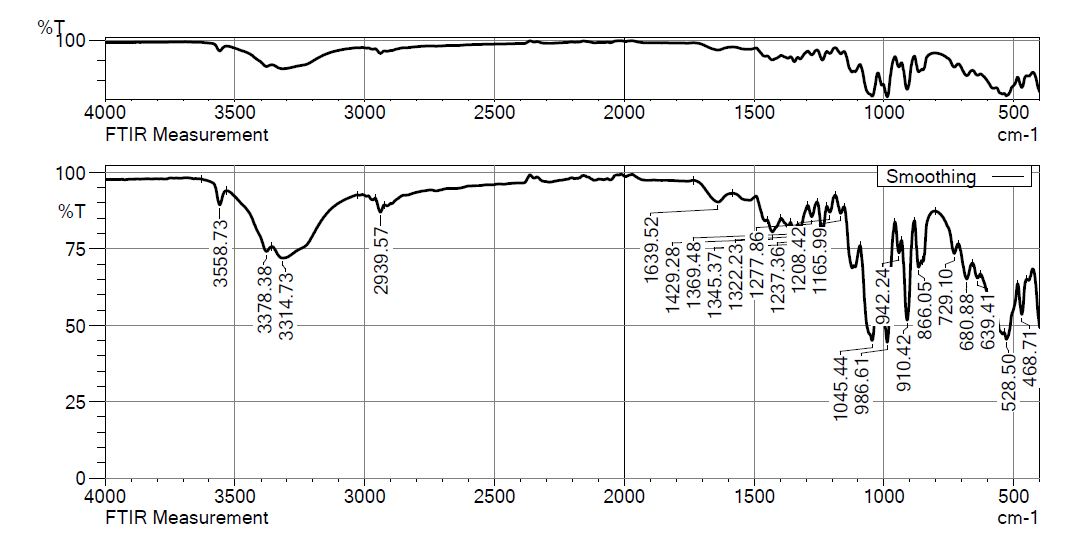


Supplementary fig. 3. FTIR of Latex


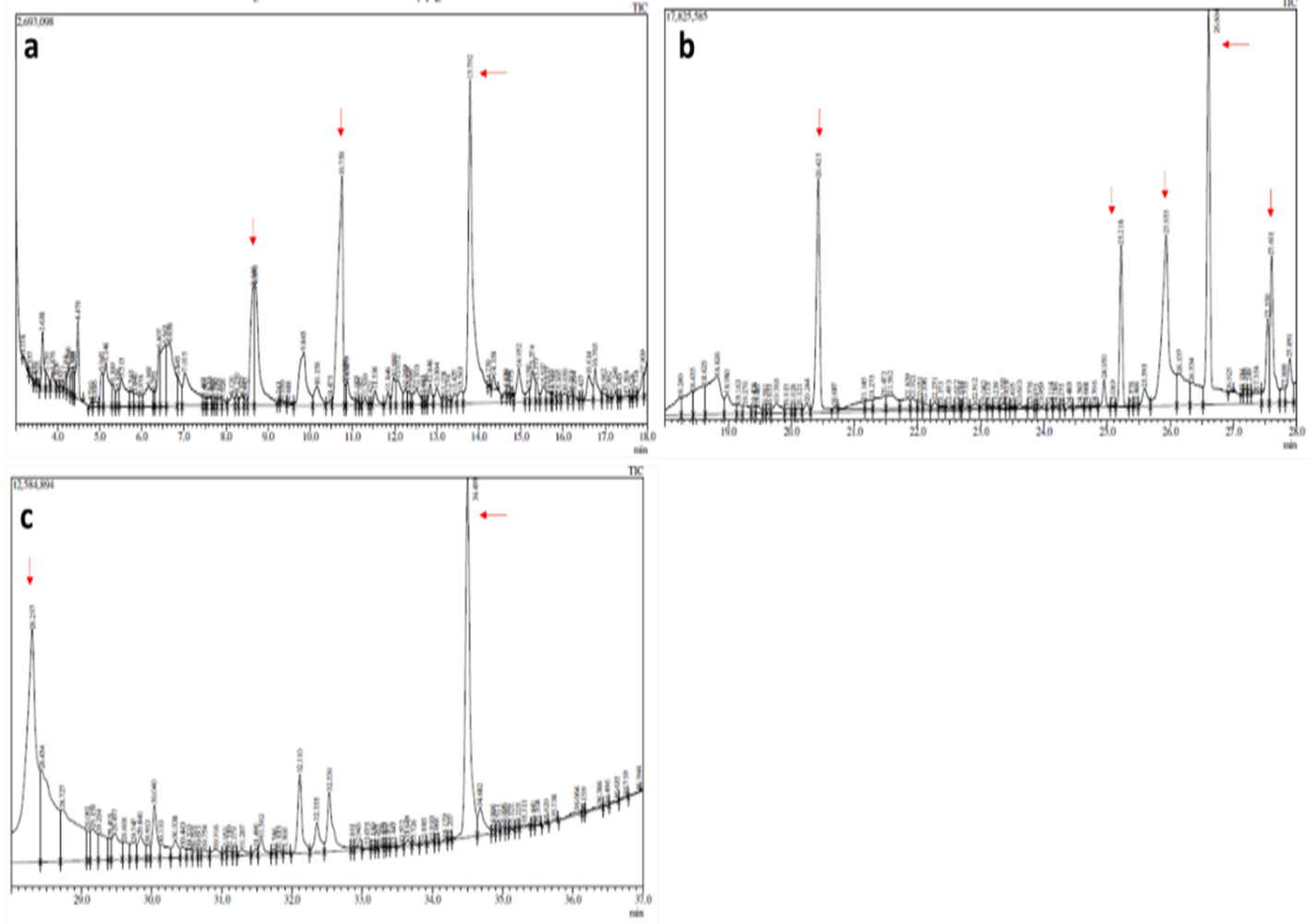


Supplementary fig. 4. GC- MS of Latex


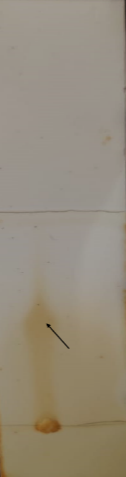


Supplementary fig. 5 –TLC of latex (Ethanol: Water - 2:1)


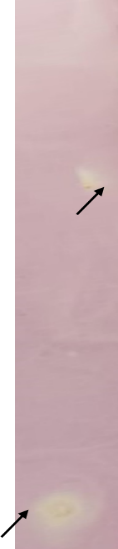


Supplementary fig. 6 –TLC DPPH bio- autography of latex (Ethanol: Water - 2:1)

Supplementary fig. 7. Percentage removal of methylene blue using latex coated SPIONs

Supplementary fig. 8. Optimization of latex coated SPIONs concentration for methylene blue removal

Supplementary fig. 9. Optimization of pH for latex coated SPIONs against methylene blue removal

Supplementary fig. 10. Optimization of Contact time for latex coated SPIONs against methylene blue removal

Supplementary fig. 11. UV- Vis of drug loaded nanoconjugate

**
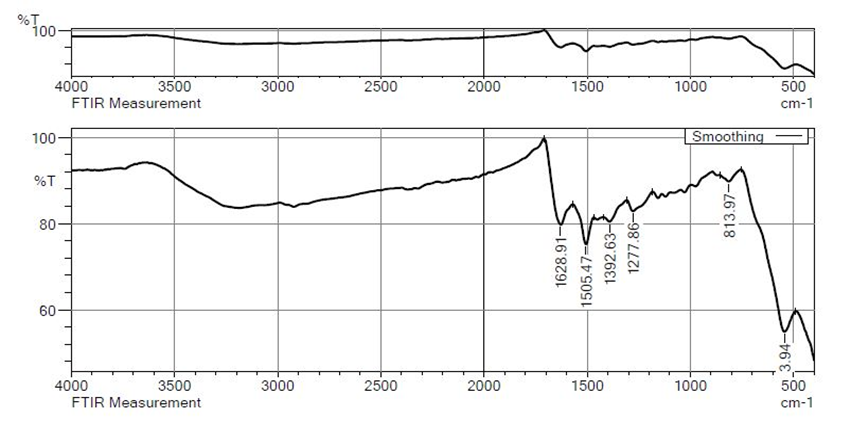
**

Supplementary fig. 12. FTIR of drug loaded nanoconjugate

**
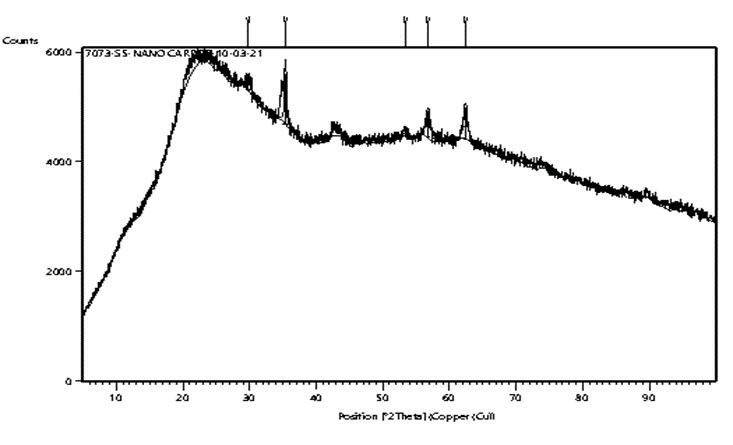
**

Supplementary fig. 13. XRD of drug loaded nanoconjugate


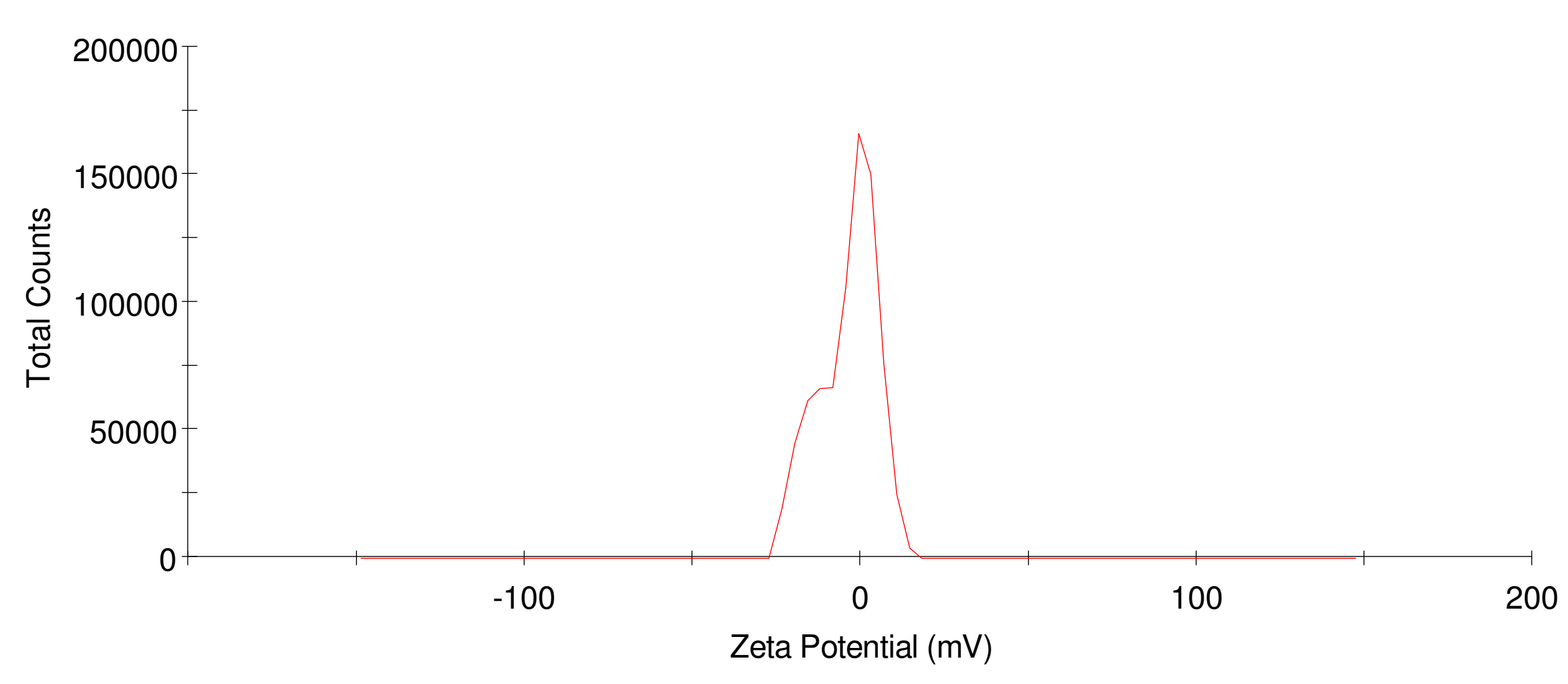


Supplementary fig. 14. Zeta potential of drug loaded nanoconjugate

**
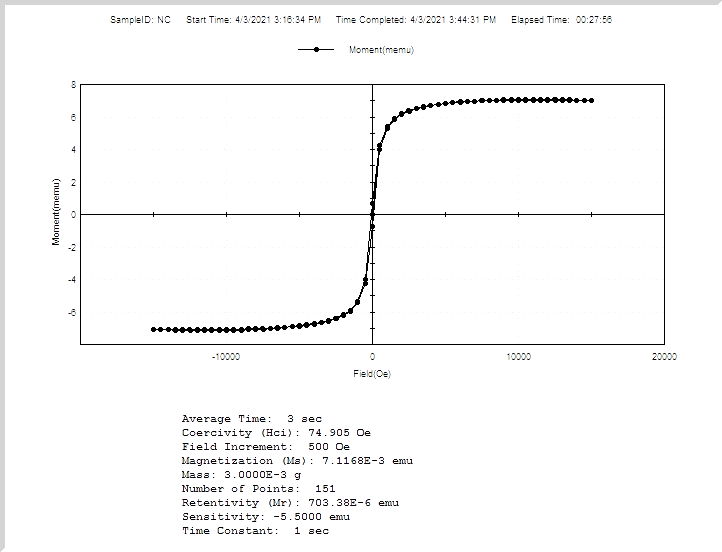
**

Supplementary fig. 15. VSM of drug loaded nanoconjugate


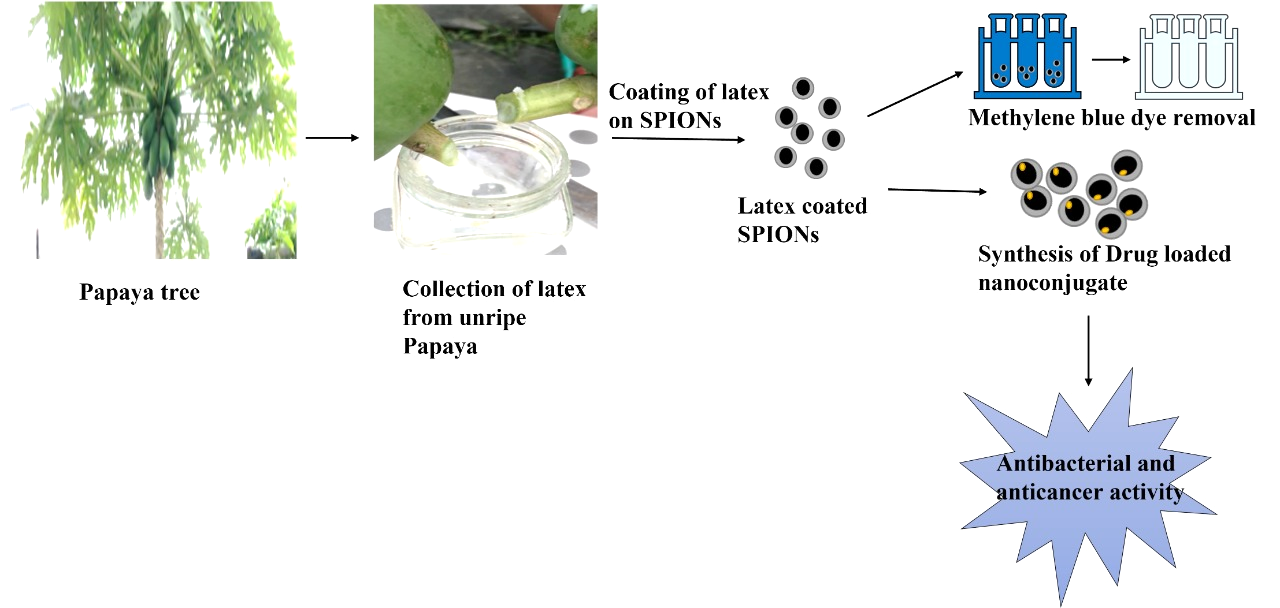


Supplementary fig. 16. Various applications of *carica papaya* latex
